# Free-breathing multi-slice myocardial T_2_ mapping using slice-selective T_2_ magnetization preparation

**DOI:** 10.1186/1532-429X-17-S1-W3

**Published:** 2015-02-03

**Authors:** Tamer Basha, Sébastien Roujol, Sophie Berg, Warren J Manning, Reza Nezafat

**Affiliations:** 1 Cardiology, BIDMC, Boston, MA, USA; 2 Harvard Medical School, Boston, MA, USA

## Background

Quantitative myocardial T_2_ mapping allows non-invasive assessment of myocardial inflammation/edema [1]. Current implementations commonly use a T_2_-prepared (T_2_prep) SSFP sequence to acquire different T_2_ weighted images at different echo times to generate the T_2_ maps [2,3]. However, all current techniques are designed for single slice acquisition with long rest cycles (3-6 sec) after each T_2_prep image acquisition to allow for full spin recovery. This markedly increases the overall scan time, especially if multiple slices are to be acquired in serial. In this study, we propose a novel *multi-slice* T_2_ mapping sequence, which uses *slice-selective* T_2_prep pulses combined with an *interleaved* slice acquisition scheme to provide a fast multi-slice T_2_ mapping.

## Methods

**Figure 1:**
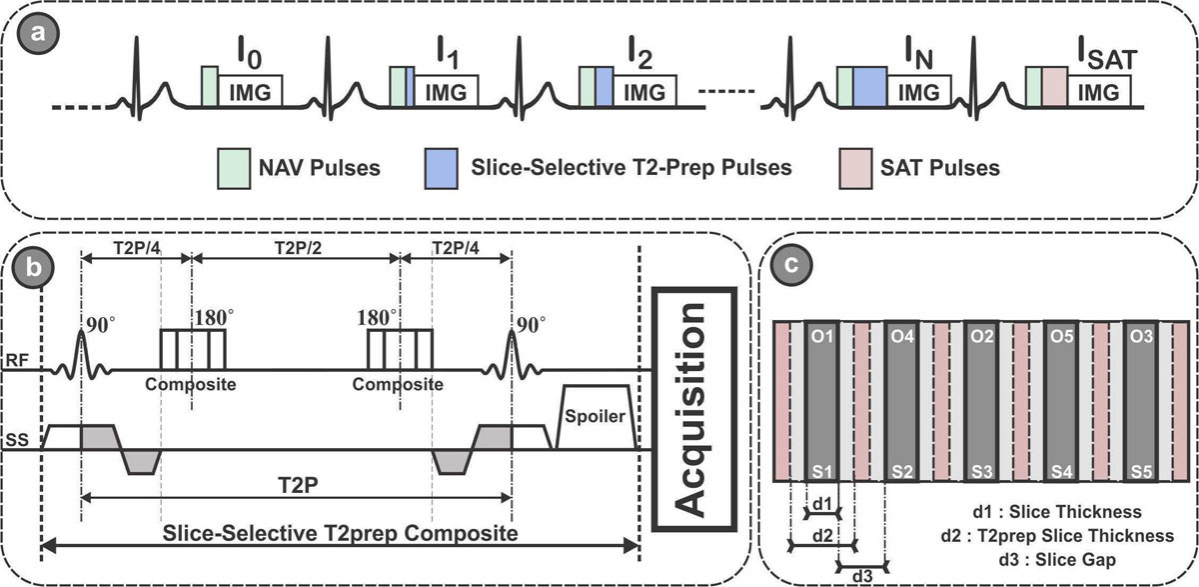
a) Schematic diagram of the proposed multi-slice T2 mapping sequence. Multiple single-shot images are acquired using ECG-triggering, following T2prep of different echo lengths, TET2P. An image, ISAT, is acquired directly after a saturation pulse to simulate the effect of a very long T2prep echo time b) The slice selective T2prep pulse consists of a tip-down slice selective 90˚ pulse, followed by four non-selective 180˚ refocus pulses (only two are illustrated in the figure) and ends with a closing tip-up slice selective 90˚ pulse. c) Slices are acquired in an interleaved fashion to allow sufficient T1 recovery time for each slice. The slice selective 90˚ pulses are applied with a slice thickness twice as imaging slice to minimize the impact of slice imperfection.

Fig. 1 shows a schematic for the proposed sequence with the proposed *slice selective* T_2_prep pulses and the interleaved slice acquisition. Upon the acquisition of a specific slice, the other slices are selectively prepared, excited and acquired during the relaxation period of that slice. Thus, one T_2_prep image is acquired at every heartbeat. Prospective slice tracking and retrospective image registration were used to correct for respiratory motion. Phantom imaging was performed using NiCl_2_ doped agarose vials, whose T_2_/T_1_ values spanned the ranges of values found in the blood and myocardium. Ten healthy adults subjects (29±17 y, 4m) were imaged on a 1.5T Phillips scanner. A free-breathing single-shot ECG-triggered slice-selective T_2_prep bSSFP sequence with the following parameters was used for acquisition of five mid-ventricular short-axis slices, FOV=320×320 mm^2^, in-plane resolution=2.5×2.5mm^2^, slice thickness=8mm, slice gap=4mm, TR/TE=2.2/1.1ms, α=40°, SENSE rate=2, acquisition window=140 ms. For comparison, a conventional breath-hold single-slice T_2_prep bSSFP sequence was performed to image the middle of the 5-slices. All acquisitions were performed using the conventional 3-images with T_2_prep echo times = 0,25,50 ms (2), with a SAT image added to compensate for the T_1_ relaxation time during readout [4]. T_2_ maps were then generated using the 3-parameter fitting model [4].

## Results

**Figure 2:**
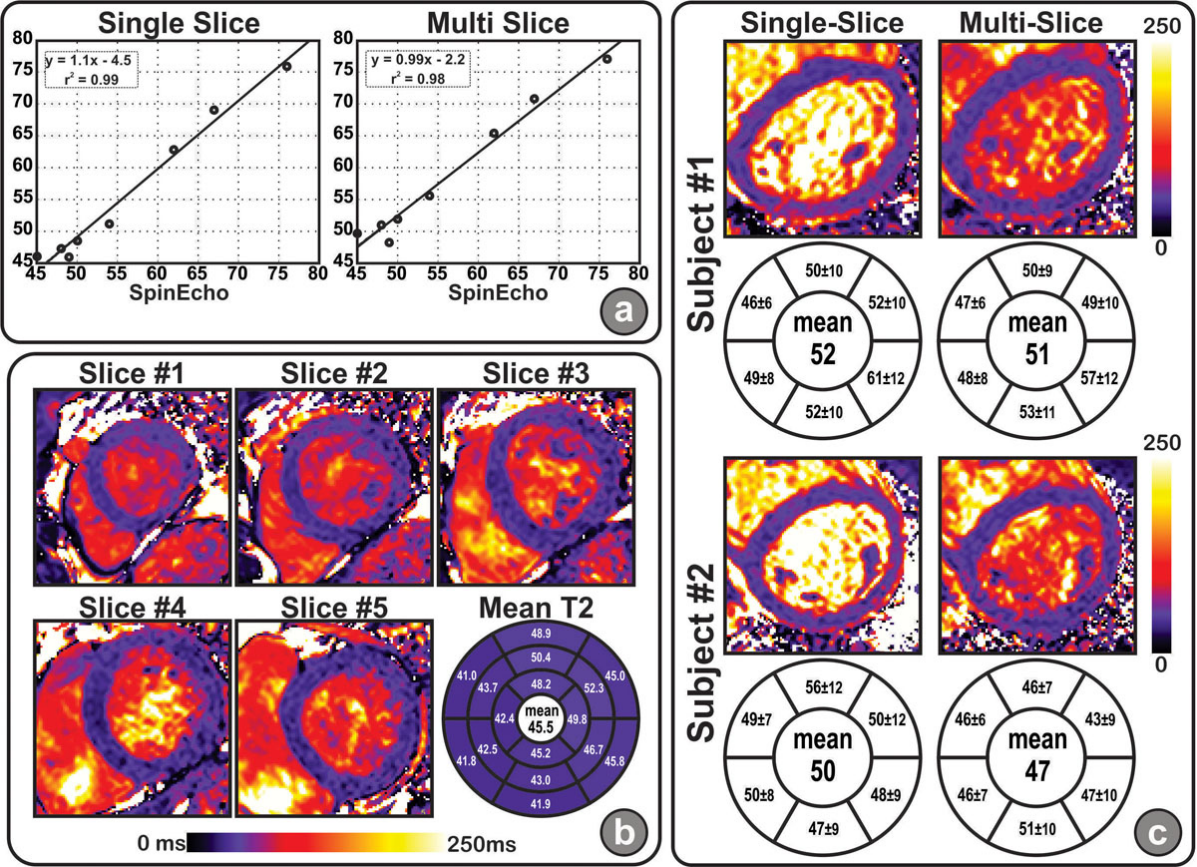
a) Correlation between T_2_ measurements measured in different phantoms using the spin echo sequence and single and multi-slice T_2_ mapping b) A representative example for the multi-slice T_2_ maps in a healthy subject. c) Two example comparisons for T_2_ maps generated using the middle slice in the multi-slice acquisition and the corresponding single slice acquisition. The bullseye shows the mean ± standard deviation of T_2_ in each segment .The center values shows the mean T_2_ for all 5 slices.

Fig. 2a shows the correlation between T_2_ measurements using the single and multi-slice sequences in phantom compared to spin echo. Figure 2b. shows an example T_2_ maps. Fig. 2c. shows a comparison between T_2_ maps generated using the single slice and multi slice sequences. The average scan time was 20 heartbeats for the 5 slices using the multi-slice and 13 heartbeats per slice using the single slice sequence. The average T_2_ across the myocardium and over all healthy subjects was 51ms and 48ms using the single and the multi-slice sequence respectively (*p*=0.1).

## Conclusions

The proposed multi-slice T_2_ mapping pulse sequence allows myocardial T_2_ measurements over the entire left ventricle by imaging of 5 interleaved slices in 20 heartbeats.

## Funding

N/A.
